# Negotiating System Requirements to Secure Client Engagement – Therapist Strategies in Adolescent Psychotherapy Initiated by Others

**DOI:** 10.3389/fpsyg.2021.704136

**Published:** 2021-09-29

**Authors:** Signe Hjelen Stige, Ingrid Eik, Hanne Weie Oddli, Christian Moltu

**Affiliations:** ^1^Department of Clinical Psychology, University of Bergen, Bergen, Norway; ^2^Department of Psychology, University of Oslo, Oslo, Norway; ^3^District General Hospital of Førde, Førde, Norway

**Keywords:** therapist behaviors, adolescent psychotherapy, client engagement, difficult therapy, therapist-client relationship, interventions, system demands

## Abstract

**Background:** Many adolescent clients come to treatment reluctantly, at the initiative of others. Adolescents also quit therapy prematurely more often than adult clients do. This points to the value of finding good ways to engage adolescent clients in treatment and understanding more of what therapists do to achieve this task.

**Methods:** We used focus group methodology to explore therapist strategies and behaviors to engage adolescent clients who come to therapy at the initiative of others. Ten focus group interviews with a total of 51 therapists were conducted with existing treatment teams from seven different clinics in community mental health care for children and youth. Reflexive thematic analysis was used as a framework to guide the analytical process.

**Findings:** Navigating a position allowing the therapist and adolescent to meet and work toward a shared understanding of the situation and what could help was considered the main gateway to client engagement. To do this, therapists had to manage the pull between system requirements and their obligation to the individual adolescent client, represented by the theme *Managing system requirements*. The process of working with the adolescent to ensure engagement is represented by the four themes: *Counteracting initial obstacles for client engagement – “You are not trapped here”; Sharing definitional power – “What does it look like to you?”; Practicing transparency – “I want you to know what I see”;* and *Tailoring as ideal – “I will design this therapy for you.”*

**Implication and conclusion:** Therapists want to understand their adolescent clients’ position better, and subsequently adjust the treatment goals and techniques to establish sufficient common ground to allow both the therapist and adolescent to find the therapeutic project worthwhile. However, system requirements and service organization were found to obstruct and influence these processes in several ways, pointing to the significance of exploring the interplay between system organization and therapeutic practice more thoroughly. There was also a variation between therapist behaviors described by different therapists within the same treatment teams, as well as systematic differences between treatment teams, pointing to the importance of future research differentiating wanted from unwanted variation in treatment.

## Introduction

Mental health problems start early and constitute a global health problem with high direct and indirect costs at an individual and societal level (Kessler et al., 2007; Steel et al., 2014). Moreover, mental health care services struggle to provide accessible and engaging treatment for adolescents. While only about 25% of children and adolescents fulfilling criteria for mental disorders receive specialized mental health care (Sadler et al., 2018), about half of those offered treatment drop out (de Haan et al., 2013). There is a potential tension between the developmental task of autonomy and the client position of needing help (Bolton Oetzel and Scherer, 2003; Radez et al., 2021; Stige et al., 2021). In addition, many adolescent clients come at the initiative of others, without feeling they need therapy (Karver et al., 2008; Stige et al., 2021). Client engagement and client-therapist rapport, key factors in therapeutic change processes, are therefore challenged in adolescent psychotherapy.

General psychotherapy research, both process-outcome research and research exploring the client perspective of therapy, has pointed to the significance of the therapist-as-person and the specific client-therapist relationship to understand therapeutic change processes (Elliott et al., 2011; Hatcher, 2015; Anderson et al., 2016; Swift et al., 2018; Heinonen and Nissen-Lie, 2020; Råbu and Moltu, 2020). Accumulating research documents therapist effects on outcomes (Wampold, 2014; Castonguay and Hill, 2017), with some therapists being more effective across a range of clients and different mental health problems. Some factors, such as the therapist’s capacity for forming an alliance with a broad range of clients, establishing a common focus for therapeutic work (i.e., collaboration), empathy, and the ability to express this in ways that make the client perceive the therapist as empathic have been established as key drivers of therapeutic change (Wampold, 2014). Moreover, the therapist’s unique contribution to these factors independent of the client’s contribution has been calculated, pointing to systematic and stable differences between therapists (Wampold and Brown, 2005; Baldwin et al., 2007; Del Re et al., 2012, 2021). In line with this, a recent literature review exploring the direct association between therapist pre-treatment factors and outcomes found that some variables in the professional domain (e.g., therapeutic attitude, professional self-doubt, and relational capacities) were associated with outcomes, while less support was found for an association between outcomes and the therapist’s private relational difficulties and social ability (Heinonen and Nissen-Lie, 2020). Moreover, some research indicates that the therapist’s interpersonal skills prior to clinical training, including the ability to communicate effectively and persuasively, the capacity to establish and repair rapport with the clients, and the capacity for empathy, have a direct association with the outcome of short-term therapy, sparking discussions on selection procedures for psychotherapy training (Anderson et al., 2016).

The significance of the initial meeting between therapist and client for the outcome of psychotherapy is reflected in the way early measurements of alliance predicts the outcome of psychotherapy across age groups (Wampold, 2014). To succeed, both in establishing and maintaining alliance and collaboration, the therapist must be flexible and sensitive, accurately assessing what is going on in therapy, accessing the client’s perspective, and adjusting the content and timing of interventions to the individual client (i.e., therapist responsiveness; Stiles et al., 1998; Hatcher, 2015; Wu and Levitt, 2020). Psychotherapy, thus, consists of unique meetings between two persons, where both parties bring with them experiences and expectations that influence the evolving interaction (Råbu and Moltu, 2020), but where the therapist bears particular responsibility – and especially so, in adolescent psychotherapy (Bolton Oetzel and Scherer, 2003).

Research from the field of adolescent psychotherapy supports the significant contribution of common factors, like the alliance (Shirk et al., 2011; Fernandez et al., 2016; van Benthem et al., 2020), with some research indicating an interaction between an adolescent’s attachment history and alliance, with the working alliance having a stronger relationship to outcomes in adolescent clients with poorer attachment histories (Zack et al., 2015). Empirical support for therapist flexibility and its impact on later client engagement and improvement in therapy has also been reported (Chu and Kendall, 2009), in line with the above-mentioned focus on therapist responsiveness (Hatcher, 2015; Wu and Levitt, 2020). Qualitative studies exploring the adolescent client’s perspective on psychotherapy similarly point to the significance of the therapist-as-person (including transparency, benevolence, and authenticity), the therapist’s management of key issues, like confidentiality, power imbalance, and client agency, and the client’s feeling of being understood, as decisive for the perceived accessibility and usefulness of therapy (Binder et al., 2011; Sagen et al., 2013; Gibson et al., 2016; Lavik et al., 2018; Løvgren et al., 2019; Radez et al., 2021; Stige et al., 2021).

Thus, there is ample evidence to suggest that establishing a good relationship and facilitating client engagement are keys to good outcomes in adolescent therapy, with therapists bearing the main responsibility for these processes (Bolton Oetzel and Scherer, 2003). We know, however, that many therapists find working with adolescent clients challenging (Everall and Paulson, 2002). The therapists’ task is made more difficult by the fact that many adolescent clients come to therapy at the initiative of others, and because the position of needing help is in tension with core developmental tasks in adolescence (Radez et al., 2021; Stige et al., 2021). Despite the key therapist task of facilitating client engagement in adolescent psychotherapy, we know very little about how therapists actually work to accomplish this difficult task (Karver et al., 2008), particularly in routine mental health care. An important local context is a development where one over the last decades have had a shift from large degrees of freedom regarding how therapists work therapeutically and how clinics organize services to more explicit national governance and a stronger focus on standardization of services (e.g., specifying services that are to be provided, requirement for coding of activity, guidelines for treatment depending on diagnosis, maximum waiting time specified depending on diagnosis). Clinics are measured on quality indicators, like waiting time, percentage of clients receiving diagnosis within the fifth session, and percentage of clients receiving medical end report within 7days of the end of treatment, and production measures, such as a clinic’s number of completed consultations is decisive for the economy of the clinic. These trends make it particularly interesting to learn more about how therapists work to facilitate the difficult clinical task of securing client engagement in adolescent psychotherapy. Hence, in the current study, we contribute to the field by exploring therapist strategies and behaviors to engage adolescent clients in therapy when others have initiated treatment, in 10 different treatment teams in routine mental health care.

## Materials and Methods

### Study Setting

This study is part of a larger study on adolescent psychotherapy in which adults initiated the process without the adolescents initially wanting therapy, consisting of individual interviews with adolescent clients and focus group interviews with therapists. Previous publications from the project include an article presenting therapists’ conceptualization of adolescent clients coming to therapy at the initiative of others (Barca et al., 2020), and one on adolescent clients’ experienced barriers and facilitators within different therapeutic trajectories (Stige et al., 2021). Therapists were found to have partly diverging conceptualizations of adolescents coming reluctantly to treatment, represented by the themes: *The hurt and distrustful adolescent; The adolescent lacking hope for the future; The adolescent engulfed in the burden of mental-health suffering;* and *The adolescent as something more than a psychiatric patient* (Barca et al., 2020). In the current article, we focus on the therapist strategies and behaviors to engage adolescent clients coming reluctantly to therapy at the initiative of others. This perspective is, however, important to see in relation to the perspective of the adolescents coming to therapy with this starting point. Analyzing 18 interviews with 12 adolescents who entered therapy at the initiative of others, we found that despite their shared starting point, these adolescents’ trajectories through mental health care differed significantly, largely relating to therapist factors, as well as system organization (Stige et al., 2021).

The study was conducted within the context of community mental health centers for children and adolescents (0–18years) in a setting where all somatic, dental, and mental healthcare are free of charge for children and adolescents. To receive treatment, a doctor/psychiatrist (most often the general practitioner), psychologist, or child protective services must send a referral. The community mental health center then assesses whether the child/adolescent’s described difficulties fulfill a right to prioritized health care, in which case the child/adolescent is offered assessment and treatment. Formally, coerced or involuntary treatment is not practiced for young people under 18, but parents/caregivers can consent to treatment on their behalf. Such cases are seen as exceptions from the general rule of consent and are individually assessed by a patient right committee. Adolescent clients may nonetheless feel pressured by parents, teachers, child protective services, or general practitioners to attend treatment.

Community mental health care in Norway is interdisciplinary, and nurses, social workers, and educators with special training are therapists under supervision, while psychiatrist and psychologists have independent diagnostic and treatment responsibility. Norway has a small population distributed over vast areas, resulting in many small clinics. Management is also clinicians, who often participate in ordinary clinical tasks. It is an egalitarian society where the power distance is low (Hofstede, 1983). National guidelines and economic incentives (e.g., payment per consultation, differentiated payment depending on assessment *vs*. treatment) regulate clinical practice, with updated commissioner’s document being provided by the government yearly.

### Design

To explore what therapists do to engage adolescents in therapy initiated by adults, we used focus group methodology. This methodology is well suited to elicit rich data on what therapists say they do, as well as to capture tension and diverging practices within and between different groups (Kitzinger, 1995; Halkier, 2002). By interviewing existing treatment teams, we were able to capture both interactions and discourses within each treatment team, as well as diverging practices between different teams. Because of the low power distance and common involvement of management in clinical tasks, team leaders and clinic leaders were viewed as valuable informants. Team leaders were included in all the focus groups, and clinic leaders were included in the focus groups in small clinics that only had one treatment team working with adolescent clients (four focus groups).

### Recruitment Procedure and Participants

We established cooperation with seven different clinics, and each clinic was given the opportunity to involve one of their clinicians as co-researchers in the project. Two clinics accepted this opportunity. We contacted clinics in different areas in Norway, from cities as well as in rural areas. Six clinics were general outpatient clinics for children and adolescents with mental health problems. One clinic had a higher degree of specialization, with one team working with adolescents with early development of psychosis, and the other team using dialectical behavior therapy with adolescents with self-harm problems and suicidal ideation. Existing treatment teams at the seven clinics were invited to participate in a focus group interview at the premises of their clinic during working hours. We conducted interviews with a total of 10 treatment teams in these clinics.

The participants in each focus group reflected the typical composition of healthcare workers in specialized mental health care for children and adolescents in Norway. Participants displayed a high degree of interdisciplinarity, including clinical psychologists, psychiatrists, resident medical doctors, psychiatric nurses, clinical special education teachers, and clinical social workers. Each focus group consisted of three to seven participants. A total of 51 participants (including team leaders and clinic leaders; 40 women) participated in the 10 interviews, with ages ranging from late 20s to late 50s.

### Data Collection

The focus group interviews were conducted between November 2017 and January 2018. The first and last author and three other interviewers conducted the interviews, with most interviews being moderated by two researchers. Each focus group interview lasted approximately 60min. Because we interviewed established treatment teams, participants knew each other and needed less time to warm up to each other. Because management was included in the focus group interviews, interviewers were particularly attentive to sign that participants did not feel free to speak their mind. Interviewers experienced that it was easy to establish rapport with the treatment teams, and conversations were rich and flowed naturally, needing little facilitation from the moderators beyond introducing the questions that guided the interviews. We, therefore, assessed that participants felt free to share their experiences despite the presence of management during interviews.

The semi-structured interview guide was developed in close collaboration with the national youth user organization Forandringsfabrikken, an NGO working to use the experiences of adolescents with school, child protective services, and mental health care services to improve those services. The interview guide covered both therapists’ conceptualization of reluctance in adolescent psychotherapy, and exploration of therapist strategies and behaviors to engage adolescent clients coming reluctantly to therapy at the initiative of others (see Table 1 for detailed questions).

**Table 1:** Interview guide for the focus group interviews with established treatment teams.

Can you first of all tell us a little bit about how you have organized the mental health care services here at your clinic? Do you experience that you have adolescent clients that come for assessment and treatment here that do not feel the need for treatment / where others have initiated the treatment? How do you recognize them? What do you look for? What do you do when you get adolescent clients for assessment and treatment that have not had a wish for treatment? In what way do you experience that service organization supports you in this work? In what way do you experience that service organization hinders you in this work? What do you think it takes to engage more of the adolescent clients who initially do not want to come here?

The interviews carried out in Norwegian and were audio-recorded and transcribed verbatim for analysis. During the interviews, one of the moderators wrote down quotes linked to each participant, so we could differentiate the voice of different participants when transcribing the interviews, thereby obtaining a picture of the interaction between participants and diverging strategies and behaviors between different therapists within each treatment team.

### Data Analysis

The data were analyzed using reflexive thematic analysis (Braun and Clarke, 2006, 2019), using NVivo 12 (QSR International Pty Ltd, 2018) as a techichal support. Initially, the second author analyzed the data under the supervision of the first author, with a broad focus on therapist behaviors when adults initiated adolescent psychotherapy, resulting in an unpublished Master thesis with the themes: *I lower the threshold for you; I am willing to be lead, and I lead; I tailor following your measurements;* and *I see what you show me*. The first author then reanalyzed the data with a narrower focus on therapist behaviors to engage the adolescent clients when they came reluctantly to therapy. Included in this reanalysis was an explicit focus on converging and diverging practices within and between treatment teams. The first author went through all transcripts and coded segments of the text detailing therapist strategies and behaviors to engage adolescent clients. Examples of diverging strategies between therapists and teams were also coded, as well as text segments illustrating how clinic organization influencing the therapist’s work. Parallel to the coding in NVivo the first author noted examples of issues and quotes illustrating differences between therapists within the same treatment team, as well as differences between treatment teams in a separate word document. The initial coding of the data material during reanalysis of the data resulted in five tentative themes: *I want you to know what I see; I will design this therapy for you; What does it look like to you? You are not trapped here*; and *Organization of services*. Using the coded material in NVivo12 (QSR International Pty Ltd, 2018) and the word document detailing differences within and between treatment teams as a starting point, the first and second authors then had a series of phone meetings, where they critically examined the tentative thematic structure with a particular focus on checking back with the data material to ensure that there were no important aspects of the therapists’ strategies or behaviors that were left out of the thematic structure. Through our discussions, it became clear to us that despite variations and diverging rationales for their strategies, and therapists across teams described very similar ideas of what were needed to ensure client engagement, as well as converging strategies and behaviors. We, therefore, decided to integrate the variation and differences between therapists and teams represented by the codes in *Organization of services* into the presentation of the remaining four themes.

Following this initial process, the tentative findings section was sent to the last two authors. The last author had conducted three of the focus group interviews and had intimate knowledge of the data material through co-authoring the article on therapist conceptualization of adolescent clients coming to therapy at the initiative of others (Barca et al., 2020). The third author had no previous knowledge of the study. All the authors then met to discuss the thematic structure, and through a series of meetings, mail correspondence, and co-writing, the final thematic structure and presentation of findings was agreed upon. The third author’s outsider position was used actively in the last parts of the analytical process and presentation of the findings, resulting in a reorganization of the findings section. During this process, it became clear to us how the observed variation within and between treatment teams largely related to the way therapists and teams perceived and interpreted system requirements and consequently their available clinical autonomy. It also became clear that these differences had a large impact on how therapists worked with client engagement, despite using shared strategies. We thus concluded that negotiation of system requirements to ensure clinical integrity was a core clinical task, and it was a theme the therapists devoted a lot of attention to during the focus group interviews. We, therefore, reorganized the thematic structure accordingly, with the theme *Managing system requirements* overarching and contextualizing the strategies detailed in the last four themes. Quotes were kept in Norwegian until the findings section was finalized, and the included quotes were then translated to English and checked by all authors. The manuscript was then professionally language edited to ensure sufficient quality of the English language.

### Reflexivity Statement

All the authors share a keen interest in adolescent psychotherapy, and how service organization and therapists’ strategies and behaviors can influence the degree to which adolescents experience therapy as being helpful. The first and last authors are clinical psychologists (PhDs), who have been working with adolescents in therapy and participated actively in the data collection. The second author will soon be a licensed clinical psychologist and has a strong interest in therapists’ behaviors in challenging clinical encounters. The third author is a clinical psychologist (PhD), who has long experience working with a range of clients, including adolescents engaging in substance abuse and their families. We, therefore, had our own preconceptions of adolescents coming reluctantly to therapy, and the services they are offered. We believe that free therapy is a good thing but recognize that free access to treatment is not sufficient to secure treatment that is effective, or to ensure that the client experiences it as helpful. Also, compared to other health care systems, practitioners have a relatively high degree of freedom in how to conduct treatment, which may lead to an individualization of responsibility for providing therapy that is experienced as helpful by the adolescent. Moreover, given our humanistic orientation, some of the therapists’ strategies were bound to resonate more with our own preferences and values. We have, therefore, worked actively and continuously to ensure that we stay open to the experiences and behaviors described by the participants without judging them normatively, using our different experiences and slightly different perspectives throughout the research process as support. Reflexive processes have, therefore, been important throughout the research process (Alvesson and Sköldberg, 2009).

### Ethics

The project followed the ethical principles stated in the Declaration of Helsinki (World Medical Association, 2013) and was approved by the regional committee on medical and health research (2016/1384/REK Vest). All participants gave their informed consent for participation. However, as the focus group interviews were organized through the clinic leaders and occurred on the premises of the clinic within working hours, this leaves the possibility that some participants felt pressured to attend the interviews. Our experience from the interviews was that in all groups, all the participants actively participated in the discussions, although amount of the time they engaged in the discussion naturally varied between participants as they had the freedom to choose how active they wanted to be in the group discussions. We, therefore, believe that we managed to safeguard the principle of voluntary consent sufficiently within our research design, and that participant felt they could speak freely despite management being present during interviews.

## Findings

Therapists in all the treatment teams described how they, in different ways, explored and responded to the adolescents’ perspectives to establish a therapeutic project that both the therapist and adolescent client found worthwhile to invest in. Yet, therapists expressed that they experienced these clinical encounters as challenging – and as something that they would put a lot of effort into without knowing if they would succeed. Moreover, being sensitive to the adolescent’s perspective was not sufficient to facilitate client engagement. Therapists in all the treatment teams described how their clinical practice was shaped and negotiated in interaction with the systemic demands of their workplace, like focus on assessment and diagnostics, coding of activity, consultation production, and diagnosis-based treatment. Hence, a core therapeutic task was to manage the tension between the obligations and tasks defined by the therapists’ employers and the mental health care system, on the one hand, and the obligations and tasks defined by each unique therapist-adolescent encounter on the other. Our analysis resulted in five main themes, of which the first, *Managing system requirements*, details the therapists’ work to negotiate space for the clinical practice they considered necessary to help the individual client within system requirements, including variance within and between treatment teams in how they solved this task and how this influenced the perceived degrees of freedom to execute their clinical tasks. The remaining four themes cover the process and the concrete therapist strategies to facilitate client engagement and were remarkable similar across therapists and treatment teams: (1) *Counteracting initial obstacles for client engagement – “You are not trapped here”*; (2) *Sharing definitional power – “What does it look like to you?”*; (3) *Practicing transparency – “I want you to know what I see”*; and (4) *Tailoring as ideal – “I will design this therapy for you.”* Therefore, the first theme constitutes an important context for understanding and the latter four themes specifying therapist behaviors and strategies.

### Managing System Requirements

A significant clinical task when working to establish engagement in adolescent psychotherapy was to manage procedures and systemic demands, like assessment and diagnosing, in ways that allowed therapists sufficient clinical integrity and flexibility to do what they considered necessary to engage their adolescent clients in a common therapeutic project:

How do we facilitate a process where they (adolescents) manage to put into words what it is really about? (…) There is shame, feelings of failing, maybe carrying the shame of others, because they have been exposed to things. So, it is such deep and vulnerable things. And then we have to in a way live up to a system. And we do the assessment. We have to fill out these forms. To satisfy a system (Participant 3, Focus group 9).

The interplay between systemic demands of increasing standardization and focus on production, current discourses of mental health care focusing on efficient diagnosis-specific treatment, and the therapists’ clinical judgment was continuous and influenced all aspects of the therapists’ described strategies and behaviors, including their ways of talking about their own clinical practice. Many therapists described, for example, how they often found themselves doing clinically meaningful work that fell outside the system guidelines and established procedures:

Because behind those symptoms there is something else, which never came up during the mental health assessment, or with their mums or teacher, but that they are at times struggling with. And then there is no motivation for sitting there talking about the stuff that was described in the referral or that someone else wanted. They want to talk about something else, but then you have to get there (relationally), so that they will talk about it. And that is not so easy when you are sitting there working with the system and parents and teachers. And, I myself, I am sitting there with an idea of what the problem is because I have read those documents and let myself be influenced by them, not being sufficiently aware of what the adolescent really wants or is thinking (Participant 2, Focus group 9).

On one hand, the therapists expressed confidence in their own clinical judgment of what therapeutic behaviors and strategies would facilitate client engagement, also when these diverged from clinical activities valued by procedures or system requirements, like focusing on getting to know the adolescent in the first sessions to make the adolescent feel safe rather than having a narrow focus on assessment and diagnostics to ensure a diagnosis within the fifth session. On the other hand, they also described a somewhat vulnerable position, where they tried their best to maneuver within their workplace system to provide the best treatment possible, without that necessarily being recognized as “proper” therapy – neither by the system nor by the persons, they were trying to help:

When you are listening to adolescents referring to what they have been offered from our system, for example (name of service user organization) or others, right, “no, I never got any treatment,” and right, they like, and that is telling… the effort you put into pondering, maneuvering, and adjusting to, all based on your professional background and understanding, but which is not understood at this same level by the adolescents, but, but which nevertheless is fruitful, right? That is quite important (Participant 6, Focus group 4).

Therapists, thus, sought to legitimize their work in different ways. During the interviews, it seemed like some of the therapists reframed their practice to align more with system requirements by using a more production-oriented language to describe therapeutic behavior that at first glance could appear in contrast with systemic demands. Other therapists borrowed authority from guidelines or known scholars to justify their clinical practice, and underlined the importance of support from colleagues in this work: “I have spent a great deal of time trying to, how can I put this, professionalize what I am doing, right? And getting support from your colleagues is vital, then” (Participant 2, Focus group 6). There were, however, quite large differences between therapists both within and between treatment teams regarding the degree to which they felt controlled and limited by the system requirements:

Clearly, it is a goal that they…the adolescent is going to have a (therapeutic) project (they find worthwhile). (…) But this is more about getting an alliance and being able to start, because you quite quickly have to get to where it (treatment focus) qualifies for specialized mental health care (Participant 6, Focus group 3).

In some teams, team leaders had paved the way for clinical judgment governing practice, providing therapists with more room and flexibility to work with the adolescents in a way that they found appropriate:

Well, a girl I am having now, I think I have spent 3 to 4months on getting her to accept treatment here. So well, right, if we realize they need treatment and that it will be hard, we spend the time needed (Participant 2, Focus group 4).

In other teams, therapists to a larger degree struggled balancing the system requirements with their professional convictions, but managed to uphold an experienced degree of flexibility:

Well, we are a relatively goal oriented organization. It is not like, Place a kid here and see if you see something, you know. There is a clear referral with an order, and we have to figure it out (…) There are guidelines for almost anything, and if you deviate too much you have to justify it. But we are relatively autonomous with regard to the treatments we offer (Participant 1, Focus group 8).

Treatment teams also differed as to service organization, despite sharing comparable contexts and system requirements, such as covering large geographical areas. In some treatment teams, the focus on fulfilling expectations detailed in the commissioner’s document resulted in limited flexibility in services:

“When we are directed to help as many people as possible in the shortest time possible, it becomes…at the same time it is said that we should be flexible. So, we mostly get people to come here” (Participant 3, Focus group 5).

Thus, conducting clinical tasks as defined by the system *versus* making independent, clinical assessments differed between treatment teams and therapists. These aspects of the service organization had direct consequences for the therapists’ work situation in more ways than influencing available room for therapist autonomy and flexibility:

Well, the caseload is so high, so there is more to do than there are people. Because we get clients from more municipalities than we are supposed to cover. So, we are pressured on time (Participant 5, Focus group 3).

And then I think, that, like, travel distance plays a role. And this is obviously to put my foot in it, but we…I do think that we would have been able to help more (adolescents) if we had had more flexible opening hours. I think that is the case. If we had some days with extended opening hours (Participant 4, Focus group 5).

While the specific situation varied between participating clinics, the shared experience between sites was that the individual therapist spent a substantial amount of energy on navigating and negotiating between system needs, on the one hand, and the needs of the individual client, on the other hand. Despite having to negotiate continuously, the constraints imposed by the workplace system and finding different solutions to these challenges, and therapists’ general descriptions suggested they had found what they experienced as satisfactory ways to perform their clinical tasks within the perceived system requirements. A few therapists, however, reported more substantial struggles with the clinic’s service organization, experiencing that standardization interfered with their clinical practice:

Some places you meet, you meet a therapist right away, right, who first makes the assessment and then the treatment. Then you have got the contact, you have built, built the relationship during the assessment, before you make the diagnoses and start treatment. Here we are doing it a bit differently, which has its advantages, but that might be challenging for the adolescents, I think, as they will first meet someone, and then shift, meeting someone else, after the assessment phase has ended (…) Although I know the system from the inside, I do not know how I would have experienced being the one who came here, facing this. I have to say, however well I know this system. Ehm (…) So, the way the knowledge production has turned into being more and more focused on alignment and standardization, I am not so sure that this is what is going to make service users experience it as useful (Participant 2, Focus group 9).

The available degree of freedom to exercise therapist flexibility and clinical autonomy within system requirements was, hence, interpreted quite differently among treatment teams and therapists, resulting in varying room for therapists to execute their preferred clinical practice when facing the challenging task of facilitating client engagement in adolescent therapy initiated by adults. Despite these differences, the therapists’ preferred strategies and therapist behaviors when working directly with their adolescent clients were remarkably similar across treatment teams, as we will detail below.

### Counteracting Initial Obstacles for Client Engagement – “You Are Not Trapped Here”

Therapists expressed their awareness of the various obstacles to client engagement even prior to the first meeting, leading them to carefully imagine and tune into what mental health care services must look like from the perspective of adolescent clients coming to therapy at the initiative of others. The main function of this strategy was to engage the adolescents long enough for them to be able to make an informed decision on whether mental health care was something worth investing in. Part of this work was to give the adolescent some experience of control as an important counterweight to the loss of adolescent autonomy implicit in the situation. Information to counteract negative preconceptions was also stated as being important. Therapists, therefore, worked hard to get the opportunity to meet the adolescent and start influencing the adolescent’s perception of what mental health care is, and what it can offer. Therapists in different treatment teams described strategies to get a foot in the door to secure the first meeting. One treatment team had, for example, established a routine where the adolescent could come three times without a referral, to get an impression of mental health care: “So, we have established a low-threshold service (for adolescents), where you can have three consultations without a referral, just to see if it is something (of interest). And if you found it helpful, you come with a referral” (Participant 6, Focus group 3).

Another team described how they would cooperate with parents to pass on the message that the adolescent could come along without having to talk:

Participant 6: “You do not have to say anything. I can say a little bit, and you can nod or shake your head if you agree with your mother,” or, like that. So, take away that you have to talk. Because I think, many (adolescent clients) think that they have to talk. In the first session we can give some information, it does not have to be a long session. We can stop after… Do not push it too far, in a way.

Participant 1: And there is something in, if the kid thinks that when you come to mental health care you have to talk about the most difficult things, then it becomes hard. So, to get that space to allow them to see, to get to know mental health care, to feel more secure. That can help them open up, I think (Focus group 2).

Normalization was widely used across treatment teams to reduce fear and sense of isolation, and make mental health care less scary. In this process, therapists used both professional knowledge, conveyed other adolescents’ experiences, and used their own experiences or imagined reactions in order to strengthen the common-human aspects of the adolescents’ situation: “And it is acceptable to say: ‘If this had happened to me, I would have become really upset and sad. And it is unfair that it is like that for you. Damn bad luck!’” (Participant 2, Focus group 6).

Therapists also recognized the challenging situation the adolescent clients found themselves in:

To say: “I do not expect you to trust me now. You have no reason to do that. Maybe, if we talk some, get to know each other a bit, give it some time. So, if we can spend some time together you might see that I am a person you can trust.” But I do not expect it (in the beginning), because that is not normal (Participant 1, Focus group 3).

Once the first contact was secured, therapists worked to ensure that the adolescent would return for subsequent sessions. Some therapists emphasized how they would be flexible regarding changes in appointments to accommodate the adolescent’s needs. Therapists from several treatment teams also shared how they would use SMS to communicate and make appointments, as they experienced this as easier for their adolescent clients. In addition, therapists across treatment teams shared how they worked actively to counteract the adolescent’s feeling of being trapped in a sticky system they had not asked to be in contact with. One commonly used strategy was to break treatment contact down to something less overwhelming, like reducing the timeline to three initial appointments. This bought them some time, while also giving them an opportunity to show the adolescent client that they could be trusted. It also provided the adolescent with an opportunity to experience and evaluate whether mental health care had something to offer that felt relevant to them:

Then there are some adolescents who think: “Now I have to go here for a hundred years! It is so hard to come here once a week!” And then I think it is helpful to make an agreement, that we will meet three times, every second week, three sessions. Only three. Nothing more. And then we have a thought behind it, that people will continue to come here. But we have to make it manageable in the beginning too (laughter). (…) So now we have three sessions. And I feel it helps to show that you are…you have to show yourself as worthy of trust somehow (Participant 2, Focus group 9).

Therapists in several treatment teams also talked about how they would use a supplementary strategy in relation to time, when this was considered fruitful:

I want to say something in relation to the time perspective. I cannot quite say, but some (adolescents) are really looking for a person to relate to. So, I do not quite know why I say it to some, and not to others. But to some (adolescents) I say: “I am here for you. I know you are having a hard time. And some come here for half a year, others a year and some for 5 years.” Because, with some (adolescents) you just feel that they need to know that if I first invest here, I will not be kicked out (Participant 3, Focus group 1).

### Sharing Definitional Power – “What Does it Look Like to You?”

Getting a better understanding of how the adolescent client saw and experienced their everyday life, and problems were reported to be a high priority and a prerequisite for offering helpful treatment across treatment teams:

Very concretely, when you have those (adolescents) who clearly do not want to be here, I say: “I really want to hear what you think about all this. Now I will talk with the adults, I will talk to the school. But I stand no chance in providing good advice or try to contribute if I cannot talk to you as well.” And then they see: “Oh, my voice is important too.” So, I feel that works. Yes, I believe, in most cases. To clearly signal that you see that they are an important person (Participant 3, Focus group 1).

Integrated in this interest was a recognition that the adolescent’s view of the situation could differ significantly from that of parents, teachers, child protective services, or others who had initiated the referral: “To not just start from what is formulated in the referral, because often the descriptions of their problems are far, far from what this young person experiences as difficult” (Participant 4, Focus group 10). Some therapists described how they actively attempted to put aside others’ perspectives to make room for the adolescent’s understanding of the situation:

To distance yourself from everything related to parents’ wishes and teachers’ demands, or child protective services, or, yes. It is there, at the back of my head. But I do not bring it into this meeting (with the adolescent), in a way (Participant 2, Focus group 9).

Therapists used different strategies to support their adolescent clients in expressing their experiences, including using available information to make informed guesses, using assessment tools for support, and giving individualized psychoeducation:

I make a guess. I say: “Sometimes it is like this, other times it can be this and that.” And you know when it fits, because then they calm down. And then you know: “Ok, this is where we are.” Because sometimes recognition is easier than explaining in their own words. And then you can explain that and normalize it (Participant 2, Focus group 4).

Some teams also routinely involved two therapists in the intake session and quite quickly split up, so one therapist talked to the adolescent and one therapist talked to the parents to create more space for the adolescents to express their point of view freely:

In a way trying to get the adolescent to understand that we are on their side, in a way, and get a break from sitting there with the parents, who say, “Yes, but that does not work.” To get a break from those interactions, in a way. That they get an opportunity to explain freely, and that: “It is ok if you totally disagree with what mom or dad said, but now I really want to hear how you feel that things are.” Regardless of it being good or bad. But that they can speak freely without parents who try to correct them, or say “no, it wasn’t quite like that, was it?” (Participant 4, Focus group 2).

Therapists also underlined the importance of sufficient time to allow the adolescent client to influence the pace and timing of approaching the difficult things – and the importance of not forcing the process:

The adolescent may not have a clear picture of what they need help for. Sometimes you ask: “What do you need help for?” and so on, and you put them in a difficult position. Because they might experience a larger degree of chaos that is difficult to put into words. So, it is something about using time to figure it out, and giving the adolescent time to figure it out, and maybe provide some suggestions, and so on, that they can recognize themselves in (Participant 3, Focus group 8).

The exploration of the adolescents’ perspective could take different forms, depending on the therapist and treatment context. One specialized team had, for example, a predetermined period of 4–6weeks, where they tried to connect with the adolescent, provide information about the treatment, and create hope, but they also explored client motivation and possible obstacles to treatment systematically, as this was seen as a gateway to continued treatment. Their position in the treatment system differed from most other teams, as all adolescents had a therapist in routine mental health care that would take over if the team or the adolescent decided this was not the time for this particular treatment. The team subsequently used strategies, such as challenging the adolescent’s expressed motivation, or giving them homework, to get a real sense of how the adolescent understood the situation and what they wanted:

Participant 4: We sell it (the treatment) to them (adolescents), but they have to, in a way, convince us to a certain degree as well, that they want this. So, we have some strategies for that in the orientation phase where we test them a bit, by, for example, presenting counter arguments for them starting treatment. Then they have to, in a way, show that they can … argue against that, for example.

Participant 3: And then we can be transparent and say: “You know what? I am not sure this is something for you. You have to convince me, to show me that you want this. It is not enough for you to just say it.” So, we are quite clear. Ideally, we want them to come with their razor blades and hand them in before they start the treatment (Focus group 7).

### Practicing Transparency – “I Want You to Know What I See”

Therapists across treatment teams stressed the importance of being transparent and providing information. This would help the adolescent understand the organization of mental health care, what would happen during the course of treatment, the rationales for different procedures and interventions, as well as the therapist’s perspective – both regarding diagnostic and treatment assessments, and impression and understanding of the adolescent as person: “I try to sum up as we go along, what I have, how I have understood this (situation), you know, and then with both facts but also with feelings” (Participant 5, Focus group 3).

In line with this, therapists in many treatment teams talked about how unique meetings between two persons were at the core of therapy. Subsequently, they strived to facilitate the development of a real relationship, including sharing personal information, such as family situations, hobbies, and pictures of pets:

I always tell a little bit about myself. Instead of just asking “Who are you?” and things like that. So, the imbalance does not get too great. That we get to know a lot (about them), and they get to know nothing (about us; Participant 2, Focus group 4).

The focus on transparency and information often rested on the therapists’ understanding of their insider-position in a system that might appear unfamiliar, confusing, and scary from the outsider-position of the adolescent client:

I think a lot of those coming here, they do not know a lot about these systems. And they know little about what governs us (therapists), and why… Well, because, for us this is so natural and given. We do the things we do and understand straight away. But, I am not so sure they (clients) understand it equally well (Participant 5, Focus group 9).

Therapists’ openness on their perspective was also seen as the main gateway to providing the adolescent client with a feeling of being understood:

I think that if they feel understood, and if they feel you see what they struggle with, it is easier to get them to come back. Or they will think: “Wow, I can get something from this.” (Participant 6, Focus group 2).

Finally, therapist transparency was thought to facilitate the development of trust, including faith in the therapist’s management of confidentiality: “‘We do not do anything without you knowing about it,’ and ‘We will talk about this,’ and try to secure them on those parts. That is how I do it” (Participant 4, Focus group 4).

### Tailoring as Ideal – “I Will Design This Therapy for You”

Therapists consistently expressed that they wanted to provide treatment that fit the conceptualization of problems and were adjusted to the adolescent’s needs and life situation. The goal was that the therapist and adolescent client both were committed to the same, therapeutic project: “That they, in a way, commit themselves and say: ‘Ok, I sign on this. This will be a project between the two of us’” (Participant 2, Focus group 4).

However, therapists also reflected on situations where facilitating client engagement no longer would be their goal:

And it is like…to say it a bit brutal, maybe not everyone is supposed to come here. It has to be ok to say “no thank you” if you really have explored how that person experiences it and what is difficult. It is really important not to medicalize someone who does not think anything is wrong. And something to do with…there are more chances at another time, if they should reach a different conclusion (Participant 5, Focus group 5).

The flexibility in tailoring the treatment was expressed in different ways. Some treatment teams traveled, for example, to enable adolescents to receive treatment without missing too much school:

We have focused on adjusting ourselves (services) and cooperation. I think about those (adolescents) attending school in X, and absence from high school, and that it is easier that we borrow an office down there. It has to do with reaching and keeping those (clients) attending high school (Participant 6, Focus group 3).

Therapists were also open to the range of approaches that could be useful for adolescent clients, including different approaches to psychotherapy, psychotherapy in combination with music therapy, or individual therapy in combination with systemic work:

When I experience that I do not succeed in talking, there is nothing to talk about, there are difficulties motivating (the adolescent) to come back, then I say: “Would you consider something to do with music?” right? And then connect them with participant 1, who is much better at that. And it is not always everyone who has a lot of words for their inner world. But then there are other options, and that is really good (Participant 4, Focus group 3).

Related to this was the experienced benefit of therapist flexibility, with each therapist managing a broad range of approaches:

A lot of us have a more eclectic education from way back, a typical X-university profile. So, if I have an adolescent, that might have a trauma history, I can work with that in one period, in another period we work on the phobia for buses, and we are outside, practicing on taking the bus, right? So, we have a lot of tools in our toolboxes, and use them depending on the phase they (adolescents) are in. I am…in a different period we might work with the aggression toward the father and the despair and anger and fear of that strong anger. So, we try…we cover a broad range, and I feel that is a good thing. I do not have to refer to someone else when it is time to treat the anxiety. I know how to do that, right? (Participant 3, Focus group 1).

## Discussion

Focus group interviews with 51 therapists in routine mental health care identified therapist strategies and behaviors to engage adolescent clients who came to therapy at the initiative of others. These strategies and behaviors were, in different ways, aimed at paving the ground to allow the adolescent’s understanding and experience of the current situation, meaningful treatment goals, etc., informing the therapists’ clinical decisions. This work was above all relational and ever evolving to adapt to the individual adolescent client. Therapist strategies and behaviors, including *counteracting initial obstacles for client engagement, sharing definitional power, practicing transparency*, and *tailoring as ideal*, reflected how they emphasized client agency and acknowledged the adolescent client as an active agent in shaping the outcome of therapy. Therapists also described how *managing system requirements* was a key clinical task, crucially influencing the available space to practice adolescent therapy in a way that engaged the individual young person, and as such, constituting an important context for their therapeutic strategies and behaviors.

**Figure 1:**
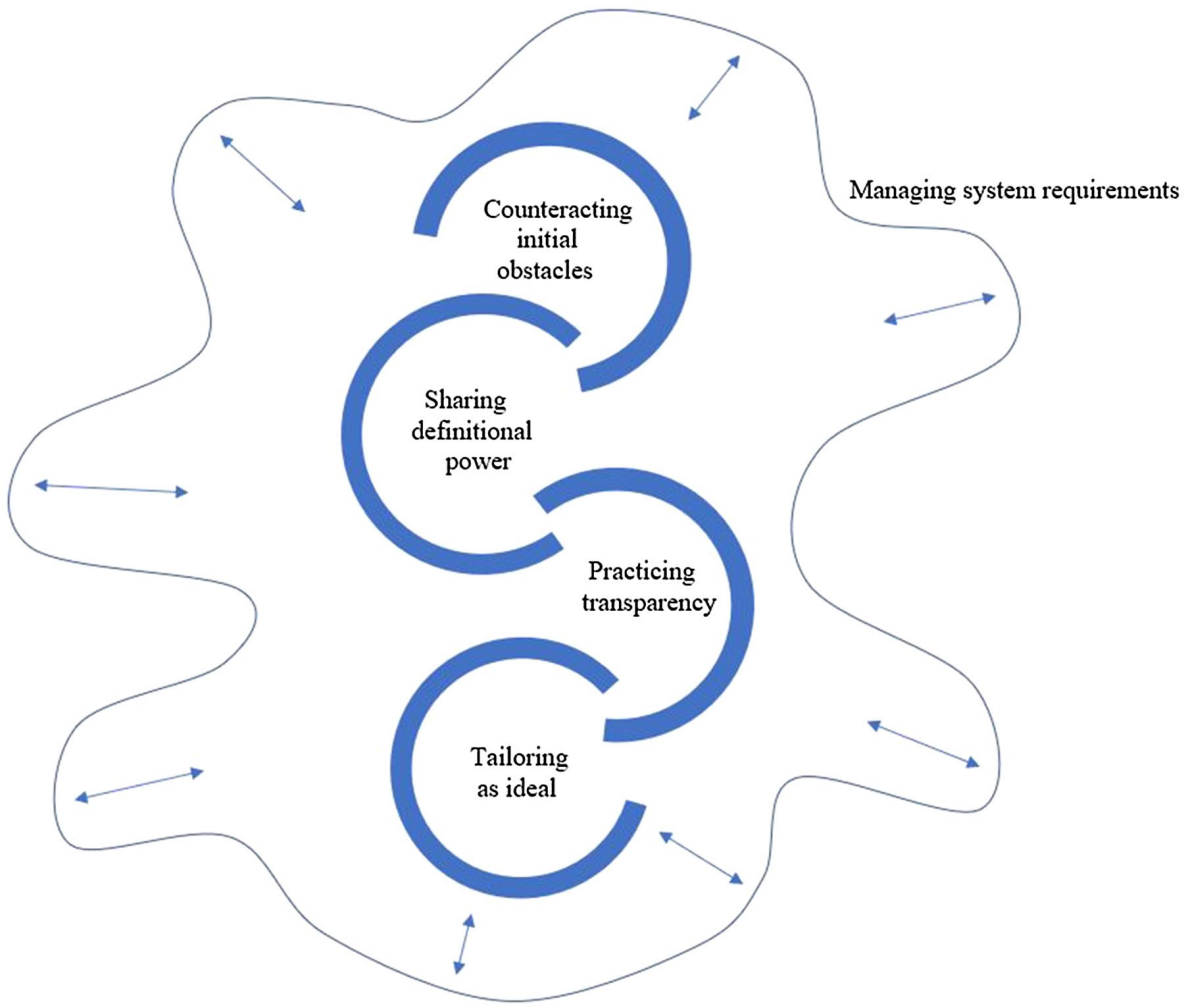
Illustration of how theme 1, *Managing system requirements*, influence available therapeutic room to work with processes facilitating client engagement.

As illustrated in Figure 1, therapists’ activities and adaptations both contribute to and are reactive to the context in which they occur. System requirements and service organization often challenged the therapists’ work, and they had to find strategies and actions to balance conflicting requirements without losing the integrity of their clinical work. We suggest that this is one potentially important nuancing contribution from our study in a field where therapist factors are often portrayed as personal traits or skills rather than contextual phenomena. Relatedly, we found diverging practices within and between treatment teams of what fell within the therapist role and tasks in mental health care. This resulted in variations in the adolescent client’s position to influence their own therapy. An implication, we would argue, is that how well a therapist manages to navigate systemic demands and requirements, and the system’s responsiveness to therapists’ constructive autonomy are factors relevant to clinical outcomes.

The themes in our study describe therapists’ actions toward securing adolescent engagement that would be in line with recommendations summarized as helpful therapeutic principles (Castonguay and Beutler, 2006). Research has firmly demonstrated that there are robust associations between relational elements, like alliance, empathy, collaboration, positive regard, goal consensus, and outcomes in psychotherapy. Other relational elements, such as congruence and the real relationship, are deemed to be probably effective. Moreover, tailoring the therapy to specific client characteristics enhances the effectiveness of psychological treatment (Gelso et al., 2018; Kolden et al., 2018; Norcross and Lambert, 2018; Norcross and Wampold, 2018). Our findings support these basic tenets and expand understanding by providing concrete descriptions of how the processes and choices are experienced during clinical work with reluctant adolescents.

One important perspective in understanding the reported findings relate to the way the problematics of the adolescents influence the therapist behaviors and strategies employed. To the therapists in this study, a focus on diagnoses did not appear to be decisive for their clinical decisions. However, as specified in Barca et al. (2020), the therapists’ perception of the adolescent clients’ prerequisites for establishing a relationship and trusting the therapists was of great importance and influenced therapist decisions. Hence, the relational elements and responsiveness were experienced as the most important therapist strategies and behaviors when working to engage adolescent clients who come to therapy at the initiative of others, acknowledging that the starting point for this work differed between adolescent clients. Themes two and three, for example, can be seen as expressions of how therapists worked actively to relate to the adolescent client’s position (empathy), adjust services to meet their needs (responsiveness), and share definitional power to allow the adolescent client to access agency. Through their examples, we see how they work hard to imagine how mental health care and the clinical situation must be experienced from the adolescent’s perspective, and what sources of information they have available prior to entering mental health care. They, therefore, used complementing strategies to obtain the prerequisite for client engagement – namely, the first meeting between therapist and client, thus, the starting point for the development of the therapeutic relationship. The therapists’ descriptions of their behaviors and strategies also are in line with recommendations for enhancing client engagement in adolescent clients (Bolton Oetzel and Scherer, 2003) and show high degrees of sensitivity to known barriers to seeking and receiving mental health care among adolescents (Radez et al., 2021; Stige et al., 2021). Yet, participants reported that their clinical priorities and preferred strategies for client engagement were under pressure.

As illustrated by the relationship between theme one and the rest of the themes in our findings, we found that participants experienced themselves to be in increasing cross pressure between standardization and individualization. For example, therapists talked about how they were expected to adjust treatment to the individual client while at the same time being measured on production indicators. These could be an expected number of client consultations that resulted in inflexibility in services. Another example is when the system pushed toward productivity and a high pace of progress, while clinical experience and judgment deemed that time and the opportunity to develop a relationship were appropriate. Several therapists also shared how they experienced that the system steered their focus and priorities in a different direction from their clinical judgment. They related how they tried to navigate these conflicting perspectives, for example, by blocking out certain periods in their calendar to make sure they had time to see their adolescent clients.

Interestingly, our findings illustrate how the same system requirements and guidelines were interpreted very differently within local contexts. This seemed largely to depend on the team leaders’ experience of agency and the degree of freedom to organize services. The findings, thus, concur with literature pointing to the ways developments, on a political or legislative level (e.g., the formulation of treatment guides and system requirements), also provoke practice modifications (Norcross and Lambert, 2018). Moreover, the ways the individual therapists and different treatment teams handled this cross pressure resulted in significant differences in the flexibility of services offered to the adolescents – thus, having real consequences for the premises of therapy for both the therapist and client.

Our findings address the concept of therapist flexibility, albeit within locally specified boundaries. Therapist flexibility is not necessarily a good thing in itself, but it can be vital for good outcomes when used judiciously (Norcross and Wampold, 2018). Kendall and colleagues attempted to integrate this knowledge and bridge the gap between manualized treatment and therapist responsiveness using the phrase “flexibility within fidelity” (Kendall et al., 2008; Kendall and Frank, 2018). This articulation and illustration of adjustment to client characteristics being a natural part of the therapeutic process, also when using treatment protocols, are an important contribution to build bridges between positions of technique *versus* relationship within the field of psychotherapy. However, our findings illustrate how this room for flexibility is not necessarily included in steering systems and legislators’, politicians’, and leaders’ understanding of what effective psychological treatment entails. This is also supported by the infrequent reliance on treatment manuals in routine clinical practice (Becker et al., 2013). This observation points to the importance of establishing reciprocal communication between clinicians and researchers, so that valuable clinical observations and experience can be utilized in research and the development of standardized treatment methods, so that clinicians feel that researchers and treatment manuals reflect the clinical reality they face (Chambless, 2014).

At the center of all this, then, is the therapist as person. While randomized controlled trials often use standardized treatment manuals to minimize the therapist’s influence, research has repeatedly demonstrated that who delivers the therapy matters (Norcross and Lambert, 2018, p. 306). This research focus on the person of the therapist also resonates with the adolescent perspective on psychotherapy, where the therapist’s interest, engagement, respect, benevolence, and sensitivity to the power imbalance are clear relational facilitators, along with the therapist’s genuineness, transparency, and flexibility (Sagen et al., 2013; Gibson et al., 2016; Lavik et al., 2018; Løvgren et al., 2019; Stige et al., 2021). However, given the context of the current state and dilemmas present within the field of psychotherapy research and the complex interplay between clinical autonomy and regulation of clinical practice in mental health care, the therapist can be seen left in an in-between position. Our findings suggest that they are left with a lot of individual responsibility for facilitating efficient psychotherapy without the corresponding degree of freedom to practice clinical autonomy. As a result, the individual therapist increasingly has to bridge and translate the clinical work and judgment made in the clinical encounters into the system requirements and control systems regulating clinical practice. It seems, therefore, important to look at how therapist-as-person, relational elements, and responsiveness can be included alongside treatment method in the conceptualizations underpinning regulations and system requirements in mental health care, thereby expanding the therapeutic room available for therapists to navigate when making clinical decisions in routine mental health care.

## Strengths and Limitations

While client engagement is difficult to achieve and important for outcomes in adolescent psychotherapy, we have limited knowledge of what therapists do to achieve this clinical task in the context of routine mental health care. This study provides descriptions of concrete therapist behaviors and strategies from a broad range of treatment teams, operating in different contexts (rural/urban, geographical distribution, etc.), thus, contributing important knowledge to the field. Moreover, we believe that the exploratory aim of the study is important, as this is an understudied area. However, we also acknowledge several limitations that need to be taken into account when reading the findings and assessing their transferability. First, we have no information about what the therapists actually did when working with their adolescent clients; we only have their descriptions of their clinical practice illustrated by numerous, detailed examples from this practice. A design in which additional data sources were included would have enabled us to expand and nuance our knowledge on the phenomenon under study by providing perspectives that contextualize the therapists’ experiences and stories. Although a common challenge for qualitative interview studies, there is important knowledge regarding a phenomenon that is not available to us through retrospective interviews alone. We believe that triangulation of data sources from within the same epistemological position is valuable, as they may provide opportunity to develop and deepen knowledge. One way to do this in a qualitative interview study could be to use interpersonal process recall of treatment sessions to elaborate on the participants perspectives on their own evaluations, choices, and behaviors as they play out in practice (see, e.g., Kleiven et al., 2020). In this study, we do not have access to other data sources, which we consider a limitation.

Moreover, while we found several differences in practice between therapists and treatment teams, the exploratory design of this study did not allow us to differentiate wanted from unwanted variation in our findings. This will be important to explore in future research. Also, although valuable informants, the decision to include management in the focus groups potentially made it more difficult for therapists to speak freely. Although we deemed that participants felt free to speak their mind, this design might have influenced the findings thus having implications for the transferability of the findings. Moreover, all the participants worked in the same healthcare system in the context of a strong welfare system, where treatment is free of charge for children and adolescents. The availability of free mental health care for children and adolescents when problems are deemed to fall within a clinical range probably increases the likelihood of meeting adolescent clients not motivated for treatment, compared to contexts where families have to pay for treatment. This has implications for the transferability of the findings.

## Conclusion

Our analysis of focus group interviews with 10 treatment teams in routine mental health care yielded five main themes, illustrating how therapists work with adolescents to achieve a position where they construct a shared understanding of the situation and what could be helpful. Therapists’ actions toward securing adolescent engagement are in line with recommendations summarized as helpful therapeutic principles. However, in implementing these principles, therapists found themselves on different levels of agreement with what was expected of them from their employers. An important finding was, therefore, how system requirements and service organization often challenged the therapists’ work, and how finding strategies and actions to balance conflicting requirements while maintaining clinical integrity was a key clinical task when working toward client engagement in adolescent psychotherapy. Our findings suggest that each therapist is left with the responsibility for facilitating efficient psychotherapy without the corresponding degree of freedom to practice clinical autonomy. As a result, the individual therapist increasingly has to bridge and translate the clinical work and judgment made in the clinical encounters into the system requirements and control systems that regulate clinical practice. Our findings, thus, provide nuance to the conceptualization of therapist-as-person and therapist effects beyond personal traits or skills, by showing how this is also a contextual phenomenon. Moreover, the therapists and treatment teams found different solutions to handle the cross pressure reflected in diverging practices within and between treatment teams of what fell within the therapist role and tasks in mental health care. This resulted in significant differences in the flexibility of services offered to the adolescents – thus, having real consequences for the premises of therapy for both therapist and client. An important implication of our findings is, therefore, that how well a therapist manages to navigate systemic demands, and the system’s responsiveness to therapists’ constructive autonomy are factors relevant to clinical outcomes. It seems important to look at how therapist-as-person, relational elements, and responsiveness can be included alongside treatment method in the conceptualizations underpinning regulations and system requirements in mental health care to facilitate therapists in their work to establish client engagement in adolescent psychotherapy.

## Data Availability Statement

The datasets presented in this article are not readily available because the data set consists of interview data, confidentiality cannot be safeguarded. Therefore, the data will not be made available. Requests to access the datasets should be directed to Signe.Stige@uib.no.

## Ethics Statement

The studies involving human participants were reviewed and approved by Regionale komiteer for medisinsk og helsefaglig forskningsetikk, Region Vest. The patients/participants provided their written informed consent to participate in this study.

## Author Contributions

SHS is the project leader and initiated the project. She has been active in all phases of the project, including design, data collection, data analysis, and writing. IE has been active in the data analysis and writing of the article. HWO has been active in the final phases of data analysis and in writing the article. CM has been active in design, data collection, the final phases of data analysis, and writing the article. All authors contributed to the article and approved the submitted version.

## Conflict of Interest

The authors declare that the research was conducted in the absence of any commercial or financial relationships that could be construed as a potential conflict of interest.

## Publisher’s Note

All claims expressed in this article are solely those of the authors and do not necessarily represent those of their affiliated organizations, or those of the publisher, the editors and the reviewers. Any product that may be evaluated in this article, or claim that may be made by its manufacturer, is not guaranteed or endorsed by the publisher.
